# Serum kynurenine levels are a novel biomarker to predict the prognosis of patients with hepatocellular carcinoma

**DOI:** 10.1371/journal.pone.0241002

**Published:** 2020-10-21

**Authors:** Shigemune Bekki, Satoru Hashimoto, Kazumi Yamasaki, Atsumasa Komori, Seigo Abiru, Shinya Nagaoka, Akira Saeki, Tomoyuki Suehiro, Yuki Kugiyama, Asami Beppu, Tamotsu Kuroki, Minoru Nakamura, Masahiro Ito, Hiroshi Yatsuhashi

**Affiliations:** 1 Clinical Research Center, National Hospital Organization Nagasaki Medical Center, Omura, Nagasaki, Japan; 2 Department of Viral Hepatitis, Nagasaki University Graduate School of Biomedical Sciences, Nagasaki, Nagasaki, Japan; Nihon University School of Medicine, JAPAN

## Abstract

**Background:**

We examined serum kynurenine levels in patients with chronic hepatitis C virus infection, and the relationship between serum kynurenine and prognosis in patients with hepatocellular carcinoma (HCC) and chronic hepatitis C.

**Methods:**

We retrospectively analyzed 604 patients with HCC diagnosed between January 1999 and December 2015, and 288 patients without HCC who were seen at the National Hospital Organization Nagasaki Medical Center between October 2014 and November 2017. The association between serum kynurenine and prognosis was evaluated using the Cox’s proportional hazards regression analysis.

**Results:**

Patients with HCC had significantly higher values of serum kynurenine than patients without HCC (median: 557.1 vs. 464.2 ng/mL, p<0.001). Five-year survival rates of HCC patients with serum kynurenine ≥900 (n = 65), 600–899 (n = 194), and <600 ng/mL (n = 345) were 30.6%, 47.4%, and 61.4%, respectively (p = 0.001, log-rank test). Multivariate analysis identified serum kynurenine as an independent predictor for prognosis of HCC patients. The hazard ratio of serum kynurenine ≥900, and 600–899 compared with serum kynurenine <600 ng/mL were 1.91 (p<0.001) and 1.37 (p = 0.015), respectively.

**Conclusions:**

A high level of serum kynurenine correlated with poor prognosis of HCC. Serum kynurenine levels may be a novel biomarker to predict the prognosis of patients with HCC. The development of drugs that inhibit kynurenine production is expected to help improve the prognosis of patients with HCC.

## Introduction

### Hepatitis C infection and liver cancer

Liver cancer is the sixth most common neoplasm and the fourth leading cause of cancer death, causing about 780,000 deaths worldwide [1]. Most primary liver cancers are hepatocellular carcinoma (HCC) [2]. The main risk factors for HCC include hepatitis B virus (HBV) or hepatitis C virus (HCV) infection, alcoholic liver injury, and nonalcoholic fatty liver disease (NAFLD) [3]. Chronic hepatitis C is a major risk factor for HCC in the United States, Europe, and Japan [3].

### Prognosis of HCC

Many systems have been proposed to predict the prognosis of HCC [4]. These systems include four characteristics that are recognized to be important determinants of survival: the severity of the underlying liver disease, tumor size, tumor progression to adjacent structures, and the presence of metastasis. These features are variably incorporated into the systems [5]. The most commonly used systems are TNM classification, the Barcelona Clinic Liver Cancer (BCLC) System [5], and the Cancer of the Liver Italian Program (CLIP) Score. Biomarkers such as alpha-fetoprotein (AFP), vascular endothelial growth factor (VEGF), angiopoietin 2 or c-Kit have been shown to potentially allow for the prognostic stratification of HCC [6].

### Amino acids and cancer

Metabolome analysis including amino acid profiling is currently under consideration as an approach to cancer screening. The plasma free amino acid profiles of cancer patients have been shown to be significantly different from those of healthy controls [7], and to change during the perioperative period [8]. With respect to HCC, each of the following has been positively correlated with the risk of developing this cancer: phenylalanine, tyrosine, the phenylalanine/tyrosine ratio, glutamate, the glutamate/glutamine ratio, kynurenine and the kynurenine/tryptophan ratio [9].

### Purpose of research

Despite the correlations described above, the serum kynurenine levels in patients with HCC and the role of serum kynurenine in the prognosis of HCC remain unknown. In this retrospective study, we aim to examine the distribution of serum kynurenine levels in patients with chronic hepatitis C, together with the relationship between serum kynurenine concentration and prognosis in patients with HCV-related HCC.

## Materials and methods

### Inclusion criteria

In this retrospective study, two cohorts of patients with chronic hepatitis C were analyzed. The first group (HCC group) included 604 patients with HCC diagnosed at the National Hospital Organization Nagasaki Medical Center between January 1999 and December 2015. The second group (non-HCC group) was selected from among the patients with chronic hepatitis C who visited the hospital from October 2014 to November 2017, and consisted of the 288 patients who did not develop HCC. All patients in both cohorts were positive for HCV antibody and all patients in the non-HCC cohort were positive for HCV-RNA.

HCC was diagnosed based on typical contrast patterns as revealed by contrast-enhanced computed tomography (CT), contrast-enhanced Magnetic Resonance Imaging (MRI) or angiography, or histopathological diagnosis was performed by needle biopsy of space-occupying lesions detected in the liver. Elevated biomarkers of HCC such as AFP and DCP were also used as a reference for HCC diagnosis.

### Exclusion criteria

Patients with chronic liver disease (HBV infection, autoimmune hepatitis, primary biliary cholangitis, alcoholic liver injury, hemochromatosis, Wilson’s disease) other than persistent HCV infection were excluded from this study. Patients with medications for depression were excluded from the study because serum kynurenine levels are high in depression [10]. The number of patients with depression excluded from this study was 20 in HCC group and 3 in non-HCC group. There were no other patients with mental diseases in this study. Patients who lacked properly stored serum or from whom consent for serum storage was not obtained were also excluded.

### Sample handling and measurement

Blood was collected at the time of HCC diagnosis for patients in the HCC cohort, and at the time of introduction of direct antiviral agents for patients in the non-HCC cohort. All separated sera were stored at -20°C until used. A medical history was collected from medical records, along with blood count and biochemical examination results at the time of collection of sera. Hematology, biochemistry and tumor marker measurements were performed using automated techniques in the clinical laboratory of this hospital.

### Measurement of serum kynurenine level

Serum kynurenine values were measured using a kynurenine ELISA kit (ImmuSmol, France) according to the manufacturer’s instructions [11]. Twenty microliters of serum were used from each sample stored at -20°C in a 96-well plate competition ELISA assay. Optical density measurements were converted to concentrations according to a standard curve. The measured values were corrected according to the dilution rate of each sample.

### Ethical considerations

Informed consent for access to medical records and specimens was obtained from each patient. Written informed consent was obtained from participants at the time of serum collection. These processes and research protocols were approved by the National Hospital Organization Nagasaki Medical Center’s Ethics Committee (approval number: 29110), and they adhere to the Helsinki Declaration of 1975, as revised in 2008, and Ethics Guidelines for Clinical Research in Japan (Ministry of Health, Labor and Welfare, Clinical Research Ethics, 2008).

### Statistical analysis

In multivariate analysis, continuous variables (aspartate aminotransferase (AST), alanine aminotransferase (ALT), serum albumin, total bilirubin, prothrombin activity, platelet count, AFP, des-gamma-carboxy prothrombin (DCP), kynurenine) were divided by median or clinically relevant values. Statistical analysis was performed for differences in quantitative values using the Wilcoxon signed rank test and the Mann-Whitney U test. Survival rates were measured using the Kaplan-Meier method, and differences in survival rates were analyzed using the log-rank test. Cox proportional hazards regression analysis was performed to assess factors associated with survival. The variables used were age, gender, AST, ALT, serum albumin, total bilirubin, prothrombin activity, platelet count, Child-Pugh classification, BCLC stage, AFP, DCP, therapy, and kynurenine. Selection of variables was assessed using a stepwise selection method. P values less than 0.05 were defined as statistically significant. Data analysis was performed using SPSS ver. 24.0 (SPSS, Chicago, IL).

## Results

### Patient backgrounds

The characteristics of HCC and non-HCC patients are summarized in Table 1. The characteristics of the HCC group were as follows. The median age of the 604 patients was 70 years, and 377 (62.4%) patients were male. The numbers of patients with Child-Pugh classes A, B and C were 436 (72.2%), 146 (24.2%) and 22 (3.6%), respectively. The numbers with BCLC stages 0, A, B, C and D were 114 (18.9%), 297 (49.2%), 126 (20.9%), 39 (6.5%) and 28 (4.6%), respectively. The median AFP was 25 (range: 1–1438472 ng/mL) and the median DCP was 50 (range: 2–841700 mAU/mL). The primary treatment consisted of surgery in 119 cases (19.7%), local therapy in 216 cases (35.8%), and angiography in 219 cases (36.3%). The mean observation period was 4.1 years. There were 304 cases (50.3%) who died within the observation period and 85 cases (14.1%) who were lost to follow-up.

**Table 1:** Demographic and clinical characteristics of the patients in the present study.

Variables	HCC group	Non-HCC group	P value
Patients, n	604	288	
Age, years	70 (38–89)	68 (20–90)	0.010
Male, n (%)	377 (62.4)	109 (37.9)	<0.001
AST, IU/L	62 (10–1098)	29 (17–214)	<0.001
ALT, IU/L	51 (0–478)	25 (12–263)	<0.001
Total bilirubin, mg/dL	0.9 (0.2–17.5)	0.7 (0.2–3.4)	<0.001
Albumin, g/dL	3.7 (1.9–5.7)	4.1 (1.9–5.0)	<0.001
Prothrombin activity, %	80.9 (33.9–131.3)	88.6 (55.1–131.7)	<0.001
Platelet count, ×10^4^/mm^3^	10.5 (0.8–100.1)	15.3 (3.9–42.7)	<0.001
Child-Pugh class, n (%)			
A	436 (72.2)	―	―
B	146 (24.2)		
C	22 (3.6)		
BCLC stage, n (%)			
0	114 (18.9)	―	―
A	297 (49.2)		
B	126 (20.9)		
C	39 (6.5)		
D	28 (4.6)		
AFP, ng/mL	25 (1–1438472)	4 (1–126)	<0.001
DCP, mAU/mL	50 (2–841700)	17 (8–256)	<0.001
Treatment to HCC, n (%)			
Surgical resection	119 (19.7)	―	―
Tumor ablation	216 (35.8)		
Transcatheter therapy	219 (36.3)		
Kynurenine, ng/mL	557.1 (40.2–1770.5)	464.2 (142.9–1464.4)	<0.001
Observation period, years	4.1 ± 3.0 *	―	―

**Abbreviations**: HCC, hepatocellular carcinoma; AST, aspartate aminotransferase; ALT, alanine aminotransferase; BCLC, Barcelona Clinic Liver Cancer; AFP, alpha-fetoprotein; DCP, des-gamma-carboxy prothrombin

Values are the medians with ranges in parentheses.

*Results are expressed as the mean ±standard deviation.

The characteristics of the Non-HCC group were as follows. The median age of the 288 patients was 68 years, which was younger than in the HCC group (p = 0.010). The number of male patients was 109 (37.9%), lower than the rate in the HCC group (p<0.001). As compared with the HCC group, AST, ALT, total bilirubin, AFP, and DCP were significantly lower (p<0.001) in the Non-HCC group, although albumin, prothrombin time (PT) and platelet count were significantly higher (p<0.001).

### Distribution of kynurenine levels

**Fig 1:**
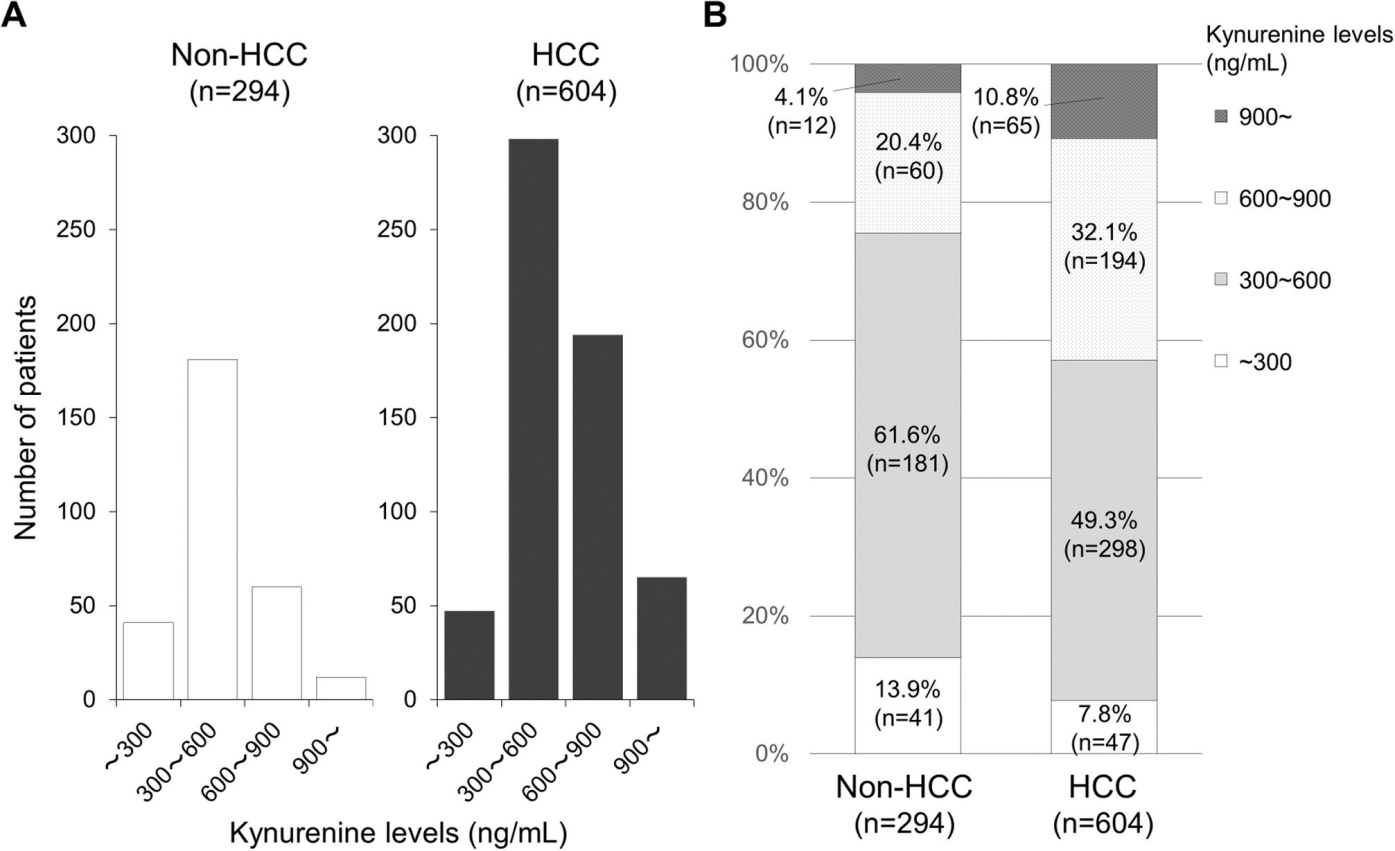
Numbers (A) and proportions (B) of patients with different kynurenine levels in HCC and Non-HCC group. (A) The median serum kynurenine level of HCC group was significantly higher than that of non-HCC group (P <0.001). (B) The proportion of serum kynurenine level of ≧600 ng/mL in HCC group was significantly higher than in non-HCC group (P<0.001).

The distribution of serum kynurenine levels in the HCC group and non-HCC group were compared (Fig 1). The median value of serum kynurenine in the HCC group was 557.1 (range: 40.2 to 1770.5 ng/mL), and that in the non-HCC group was 464.2 (range: 142.9 to 1464.4 ng/mL). The serum kynurenine value of the HCC group was significantly higher compared to that in the non-HCC group (p<0.001). The percentage of patients with a serum kynurenine value of 600 in the HCC group (42.3%) was significantly higher than that in the non-HCC group (24.5%) (p<0.001). The relationship between serum kynurenine levels and other clinical parameters was investigated, and serum kynurenine levels showed no correlation with other factors except for DCP (r = 0.207).

The HCC group was divided into three groups based on serum kynurenine levels (Table 2). The number of patients with serum kynurenine <600, 600–899, and ≥900 ng/mL were 345, 194, and 65, respectively. Compared to patients with serum kynurenine levels <600 ng/mL, patients with serum kynurenine levels of ≥900 ng/mL were older (72 vs. 69 years, p<0.01) and had lower albumin levels (3.6 vs. 3.8 g/dL, p<0.05), higher tumor marker scores (AFP: 53 vs. 22 ng/mL, p<0.001; DCP: 143 vs. 43 mAU/mL, p<0.05), and a greater percentage of patients in advanced BCLC stages (BCLC B, C, D: 50.8% vs. 26.7%, p<0.001).

**Table 2:** Clinical characteristics of the patients with HCC divided into three groups according to the kynurenine levels.

Kynurenine levels, ng/mL	<600	600–899	≧900	P value
Patients, n	345	194	65	
Age, years	69	71	72	<0.05^¶^, <0.01^‡^
Male, n (%)	203 (58.8)	129 (66.5)	45 (69.2)	0.103
AST, IU/L	62	63	59	n.s.
ALT, IU/L	53	51	44	n.s.
Total bilirubin, mg/dL	0.9	0.9	0.8	n.s.
Albumin, g/dL	3.8	3.7	3.6	<0.05^‡^
Prothrombin activity, %	82.0	77.8	82.5	n.s.
Platelet count, ×10^4^/mm^3^	11.1	10.4	10.0	n.s.
Child-Pugh class, n (%)				0.583
A	254 (73.6)	140 (72.2)	42 (64.6)	
B	79 (22.9)	46 (23.7)	21 (32.3)	
C	12 (3.5)	8 (4.1)	2 (3.1)	
BCLC stage, n (%)				<0.001
0	76 (22.0)	33 (17.0)	5 (7.7)	
A	177 (51.3)	93 (47.9)	27 (41.5)	
B	64 (18.6)	43 (22.2)	19 (29.2)	
C	13 (3.8)	15 (7.7)	11 (16.9)	
D	15 (4.3)	10 (5.2)	3 (4.6)	
AFP, ng/mL	22	28	53	<0.01^‡^
DCP, mAU/mL	43	50	143	<0.05^‡^
Treatment to HCC, n (%)				<0.05
Surgical resection	74 (21.4)	37 (19.1)	8 (12.3)	
Tumor ablation	123 (35.7)	39 (40.7)	14 (21.5)	
Transcatheter therapy	122 (35.4)	66 (34.0)	31 (47.7)	

**Abbreviations**: HCC, hepatocellular carcinoma; AST, aspartate aminotransferase; ALT, alanine aminotransferase; BCLC, Barcelona Clinic Liver Cancer; AFP, alpha-fetoprotein; DCP, des-gamma-carboxy prothrombin

^¶^ <600 vs 600–899

^‡^ <600 vs ≧900

^♭^ 600–899 vs ≧900

### Serum kynurenine was related to survival in HCC patients

We examined whether serum kynurenine levels were associated with survival in HCC patients. In multivariate analysis, age, total bilirubin, albumin, Child-Pugh class, BCLC stage, AFP, DCP, treatment (surgery), and serum kynurenine levels were independent factors related to survival (Table 3).

**Table 3:** Variables associated with mortality of patients with HCC.

Variables	Univariate HR (95%CI)	P-value	Multivariate-adjusted HR (95%CI)	P-value
Sex (1; male, 0; female)	0.92 (0.73–1.16)	0.460	0.85 (0.66–1.10)	0.227
Age ≧70 years	1.26 (1.003–1.58)	0.047	1.66 (1.29–2.13)	<0.001
AST ≧63 IU/L	1.37 (1.09–1.72)	0.006	1.04 (0.73–1.46)	0.842
ALT ≧51 IU/L	1.06 (0.84–1.33)	0.634	1.20 (0.8601.71)	0.278
Total bilirubin ≧1 mg/dL	1.94 (1.55–2.43)	<0.001	1.68 (1.27–2.24)	<0.001
Albumin < 3.7 g/dL	2.28 (1.82–2.87)	<0.001	1.43 (1.05–1.95)	0.025
Prothrombin activity < 79%	1.76 (1.40–2.21)	<0.001	0.94 (0.69–1.29)	0.711
Platelet count ≧10.5 ×10^4^/mm^3^	1.26 (1.01–1.58)	0.043	0.94 (0.72–1.23)	0.675
Kynurenine, ng/mL				
<600	1	―	1	―
600–900	1.32 (1.04–1.67)	0.021	1.35 (1.04–1.74)	0.025
≧900	2.15 (1.53–3.03)	<0.001	1.98 (1.35–2.91)	<0.001
Child-Pugh class				
A	1	―	1	―
B	2.16 (1.68–2.77)	<0.001	1.45 (0.99–2.13)	0.055
C	―	―	3.97 (1.25–12.59)	0.019
BCLC stage				
0	1	―	1	―
A	0.67 (0.54–0.84)	<0.001	1.38 (0.93–2.05)	0.010
B	1.97 (1.52–2.56)	<0.001	1.76 (1.08–2.88)	0.023
C	2.98 (1.97–4.49)	<0.001	3.65 (1.96–6.79)	<0.001
D	7.17 (4.52–11.39)	<0.001	2.17 (0.80–5.88)	0.129
AFP (per 1 log)	1.77 (1.58–1.98)	<0.001	1.33 (1.15–1.53)	<0.001
DCP (per 1 log)	1.66 (1.49–1.86)	<0.001	1.45 (1.24–1.69)	<0.001
Treatment				
Transcatheter therapy	1	―	1	―
Tumor ablation	0.69 (0.54–0.87)	0.002	0.91 (0.66–1.26)	0.581
Surgical resection	0.37 (0.26–0.52)	<0.001	0.31 (0.21–0.46)	<0.001

**Abbreviations**: HCC, hepatocellular carcinoma; AST, aspartate aminotransferase; ALT, alanine aminotransferase; BCLC, Barcelona Clinic Liver Cancer; AFP, alpha-fetoprotein; DCP, des-gamma-carboxy prothrombin

Hazard ratios for the survival rate were calculated by Cox proportional hazards analysis.

**Fig 2:**
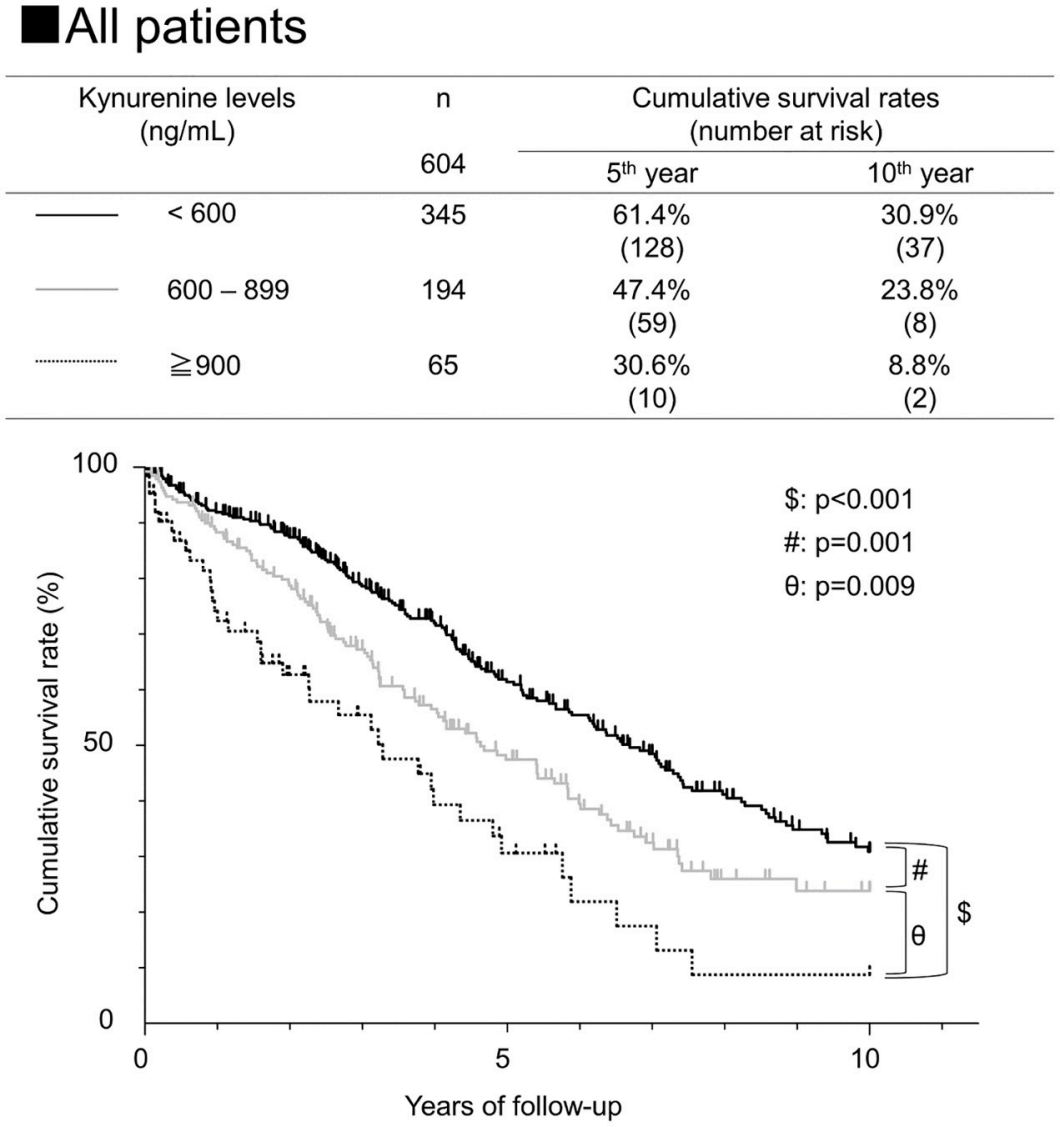
Cumulative survival rates of HCC patients according to the kynurenine levels. Cumulative survival rates of HCC patients according to kynurenine levels was analyzed using Kaplan-Meier’s method. Black solid, gray solid, and dotted lines indicate stratified kynurenine levels of <600, 600–899, and ≧900, respectively. Incidence rate differed significantly among the three groups by the log-rank test, increasing along with the kynurenine level.

When the survival rates were compared with the serum kynurenine levels, the 5-year survival rates for patients with serum kynurenine levels of <600, 600–899, and ≥900 ng/mL were 61.4%, 47.4%, and 30.6%, respectively (Fig 2). The survival rate decreased significantly with each step of the serum kynurenine levels from <600, to 600–899, and then to ≥900 ng/mL (p<0.001: kynurenine <600 and ≥900; p = 0.001: kynurenine <600 and 600–899; p = 0.009: kynurenine 600–899 and ≥900).

**Fig 3:**
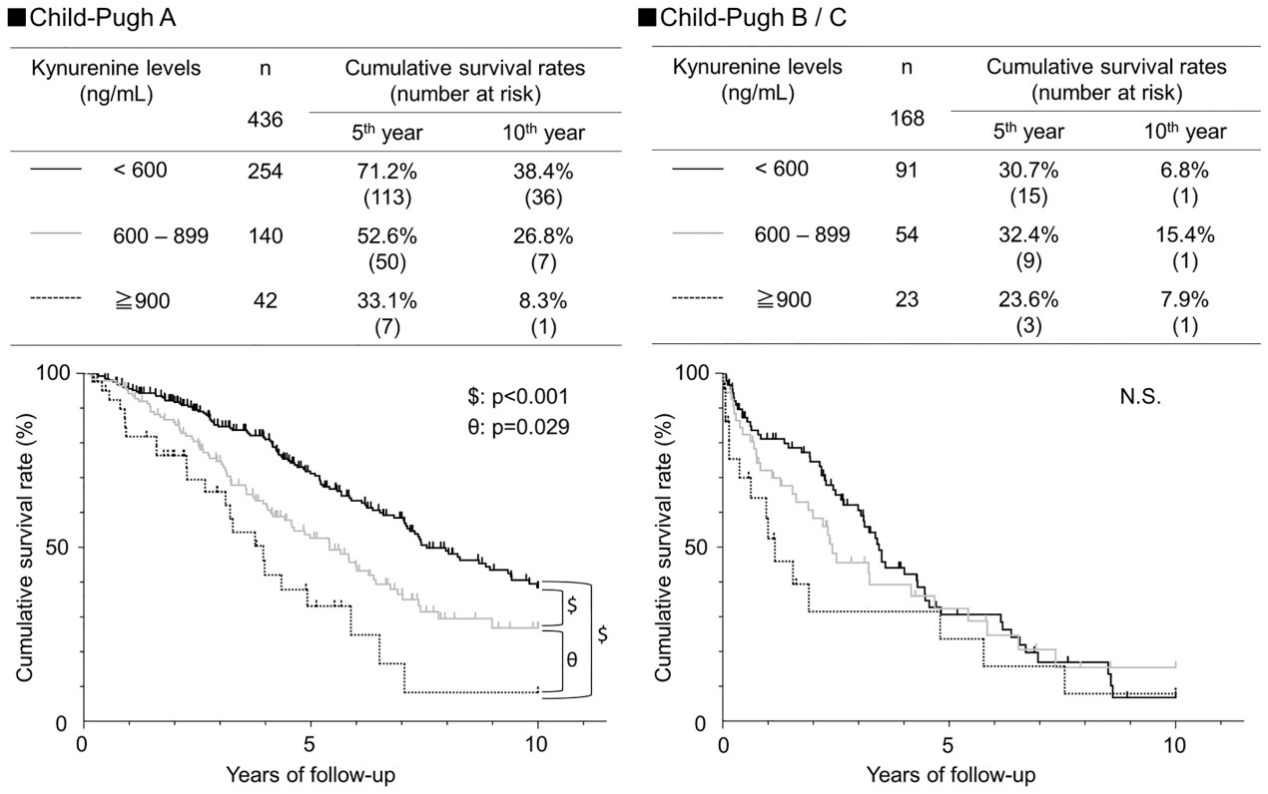
Cumulative survival rates of HCC according to the kynurenine level, stratified by Child-Pugh class. Cumulative survival rates of HCC according to the kynurenine level were analyzed using Kaplan-Meier’s method. Black solid, gray solid, and dotted lines indicate stratified kynurenine levels of <600, 600–899, and ≧900 ng/mL, respectively. In Child-Pugh A, survival rates differed significantly among the three groups by the log-rank test, increasing along with the kynurenine level.

**Fig 4:**
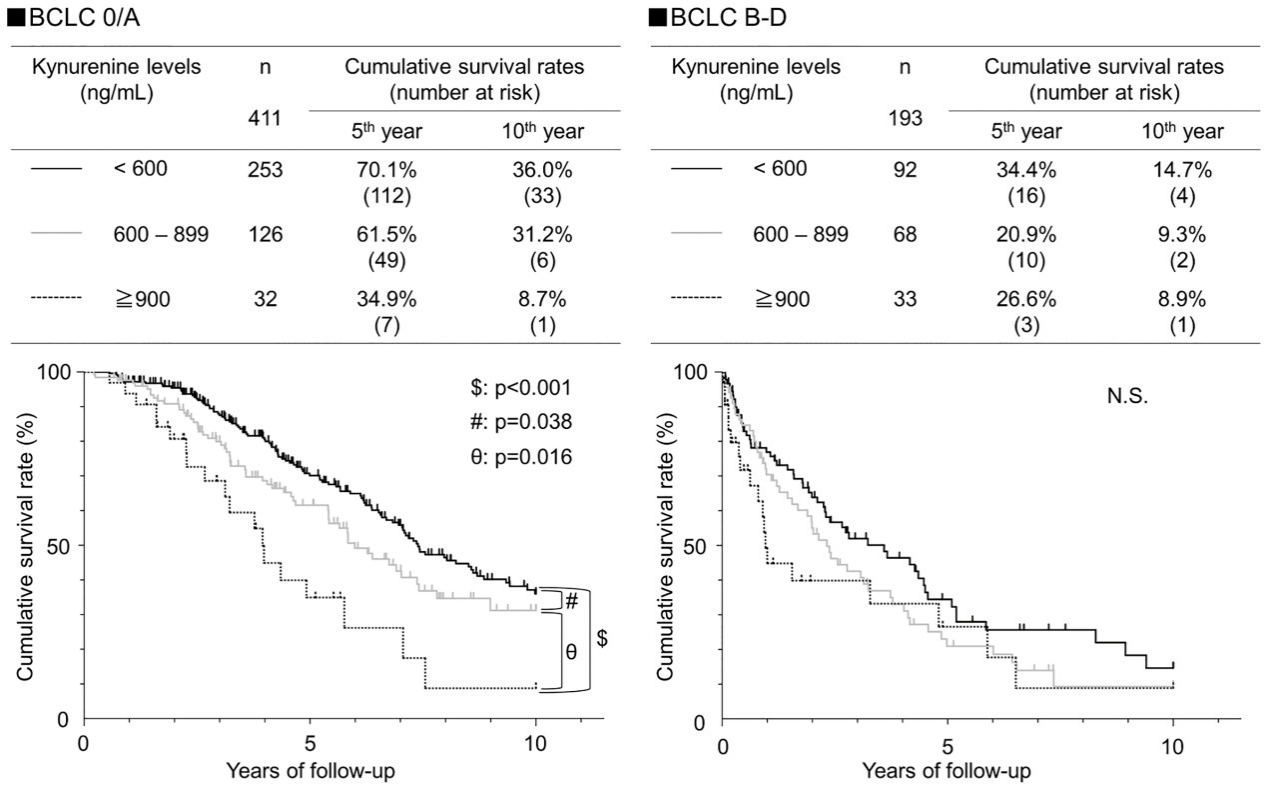
Cumulative survival rates of HCC according to the kynurenine level, stratified by BCLC stage. Cumulative survival rates of HCC according to the kynurenine level were analyzed using Kaplan-Meier’s method. Black solid, gray solid, and dotted lines indicate stratified kynurenine levels of <600, 600–899, and ≧900 ng/mL, respectively. In BCLC stage 0 and A, survival rates differed significantly among the three groups by the log-rank test, increasing along with the kynurenine level.

Fig 3 shows the relationship between kynurenine levels and prognosis, stratified by Child-Pugh class. In patients with Child-Pugh A, the 5-year survival rate was 71.2% for serum kynurenine levels <600, 52.6% for those 600–899, and 33.1% for those ≥900, and significantly different between each pair of groups (p<0.001: kynurenine <600 and ≥900, kynurenine <600 and 600–899; p = 0.029: kynurenine 600–899 and ≤900 ng/mL). In patients with Child-Pugh B or C, there was no significant association between serum kynurenine levels and prognosis. Fig 4 also shows the relationship between kynurenine levels and prognosis stratified by BCLC stage. In patients with BCLC stage 0 or A, survival rates were 70.1% for patients with serum kynurenine levels <600, 61.5% for those with serum kynurenine 600–899, and 34.9% for those with serum kynurenine ≤900, and these differences were significantly different between each pair of groups (p<0.001: kynurenine <600 and ≥900; p = 0.038: kynurenine <600 and 600–899; p = 0.016: kynurenine 600–899 and ≥900 ng/mL). In patients with BCLC stage B, C or D, no significant association was found between serum kynurenine levels and prognosis.

## Discussion

This study is the first to show that serum kynurenine is elevated in patients with HCC. The kynurenine pathway of tryptophan degradation generates several metabolites known as kynurenines. Kynurenine and its metabolites are known for their effects on the central nervous system and associated with several psychiatric and mental health disorders, such as depression and schizophrenia [12]. In this study, serum kynurenine levels in patients with type C chronic liver disease were significantly higher in the HCC group than the non-HCC group. The average value of serum kynurenine was reported to be 291.5 to 460.2 in the measurement of healthy control [13, 14], and the normal range of kynurenine is considered to be approximately this value. In patients with type C chronic liver disease, increases in serum kynurenine levels, expression of indoleamine 2,3-dioxygenase (IDO) as an enzyme producing kynurenine, and the serum kynurenine/tryptophan ratio have all been demonstrated [15, 16]. When HCC develops in chronic hepatitis C, kynurenine production is considered to be further promoted.

Another new finding in this study is that serum kynurenine was a prognostic factor in patients with HCV-related HCC. In a cohort of 604 HCC patients, Kaplan-Meier analysis of the overall survival showed that high serum kynurenine values were associated with poor prognosis in patients with HCC. Cox proportional hazards analysis revealed that serum kynurenine values were independent prognostic markers of overall survival. These findings suggest that upregulation of kynurenine may serve as a marker for identifying patients with poor clinical outcome.

It has been reported that, in hematological malignancies, the cutoff value of serum kynurenine was set and divided into 2 groups, the prognosis was poor in the group with high value [17, 18]. In addition, a report on influenza virus infection has compared the presence or absence of disease progression among the 3 groups based on serum kynurenine levels [19]. The report has shown that the higher the kynurenine value, the higher the odds ratio for disease progression. In this study, we set the cutoff value to 600, which is close to the median value of the HCC group and the cutoff value reported in the past, and also to 900 to distinguish cases with a poorer prognosis.

The current grading systems show to some extent the prognosis of patients with HCC, but due to tumor heterogeneity and accumulation of genetic and epigenetic changes, the same stage of HCC may show considerable differences in prognosis. Therefore, it would be useful to look for prognostic factors that can effectively distinguish patients with good or poor prognosis at the same stage [20]. Our results showed that high expression of kynurenine in HCC patients with BCLC stage 0 or A was closely associated with shorter survival. In addition, high expression of kynurenine in HCC patients with Child-Pugh A was also associated with shorter survival times. Thus, our results indicate the potential value of kynurenine in predicting survival in patients with early-stage HCC, and in HCC patients with preserved hepatic function. When HCC patients with BCLC stage 0 or A were divided into three groups according to the serum kynurenine levels of <600, 600–899, or ≥900, all other factors related to survival prognosis, such as age, Child-Pugh class, or tumor markers, were not significantly different among the three groups. This result suggests that kynurenine appears to affect the prognosis of patients with HCC independently of other factors. On the other hand, the prognosis was poor for C-P B, C and BCLC B-D. In these patients, tumor factors and hepatic reserve factors had a stronger effect on prognosis than kynurenine, which may be the reason why kynurenine was not extracted as a significant factor. We investigated the relationship between serum kynurenine and other factors. Serum kynurenine was correlated with age (correlation coefficient 0.175, P<0.001), serum albumin (correlation coefficient -0.106, P = 0.002), AFP (correlation coefficient 0.184, P<0.001), DCP (correlation coefficient 0.207, P <0.001). However, the correlation coefficient was relatively low in all cases, and the association was considered to be weak.

We also examined the relationship between serum kynurenine and the degree of HCC differentiation. In this study, there were 297 cases with pathological findings in the HCC group. Of these, 113 were well differentiated (median serum kynurenine level; 522.2 ng/mL), 106 were moderately differentiated (median serum kynurenine level; 509.25 ng/mL), and 35 were poorly differentiated (median serum kynurenine level; 543.9 ng/mL). The ratio of the degree of HCC differentiation was examined in the groups with serum kynurenine levels <600, 600–900, and >900. The numbers of well differentiated, moderately differentiated, and poorly differentiated cases were 68 (42.5%), 73 (45.6%), 19 (11.9%) in <600, 39 (49.4%), 26 cases (32.9%), 14 cases (17.7%) in 600–900, and 6 cases (40.0%), 7 cases (46.7%), 2 cases (13.3%) in >900, respectively. There was no significant difference in the rate of differentiation (P = 0.394).

Kynurenine is a tryptophan metabolite produced by two IDO enzymes (IDO1 and IDO2) and tryptophan 2,3-dioxygenase (TDO). High IDO or TDO levels are reported as poor prognostic factors in various malignant tumors, such as brain tumor cells [21], ovarian cancer [22], acute myeloid leukemia [23], breast cancer [24], and Hodgkin’s lymphoma [25]. A systematic review and meta-analysis of 24 studies involving 2706 patients reported shorter overall survival and worse disease-free survival in patients with high expression of IDO1 [26]. The results of our present study indicate that serum kynurenine is correlated with the prognosis of patients with HCC, and we consider that the generation of kynurenine by IDO and TDO contributes to the deterioration of the prognosis. Kynurenine is a simple biomarker because it can be easily measured in serum.

Kynurenine has been shown to be a specific agonist of the aryl hydrocarbon receptor (AhR) [27]. AhR is a transcription factor and activation of AhR of cancer cells by kynurenine increases the expression of genes promoting cell migration [28]. IDO expression can cause cell cycle arrest and apoptosis of effector T cells, and differentiation and maturation of new regulatory T cells with potent regulatory activity from undetermined CD4 + T cells [29]. Activation of the kynurenine pathway is thought to influence the enhancement of cancer cell malignancy and the mechanism of tumor immune evasion, which could account for the association between kynurenine levels and prognosis.

It was reported that intraperitoneal injection of mastocytoma cells that overexpress TDO induced potent immunosuppression, which was reversed by pharmacological inhibitors of enzyme activity, leading to immune-mediated tumor rejection [30]. Other studies established that the combination of immune checkpoint blockade and IDO1 pathway inhibition results in potent reactivation of tumor infiltrating T cells and/or tumor-resident immunosuppressive regulatory T cells [31, 32]. Currently, several IDO inhibitors are undergoing clinical trials, either as monotherapy or for use in combination with other immunotherapies and chemotherapy [33, 34]. In HCC cases with elevated serum kynurenine, IDO is likely to be activated. Therefore, it is expected that IDO inhibitors will attenuate the activity of IDO and subsequently decrease the kynurenine level, leading to an improvement of the prognosis of HCC patients.

Certain limitations of this research bear mention. First, this was a retrospective analysis, and thus future prospective analysis would be warranted. In addition, metabolites of kynurenine pathways other than the kynurenine pathway were not measured in this study. Additional studies will be needed to examine whether other metabolites have a relationship with HCC. In this study, inclusion period between HCC patients and non-HCC patients were different. To predict the prognosis of HCC patients, we set a long observation period in HCC group. On the other hand, in non-HCC patients, only a cross-sectional observation was performed and a sufficient number of cases could be incorporated in a short period of time, therefore a different observation period was set.

In conclusion, it has been shown that serum kynurenine levels can be a novel biomarker to predict the prognosis of patients with HCC. We expect that the development of drugs that inhibit kynurenine production will help to improve the prognosis of patients with HCC.

## Supporting information

S1 Dataset Anonymized data set. (XLSX)
